# An Efficient Rescue System with Online Multi-Agent SLAM Framework

**DOI:** 10.3390/s20010235

**Published:** 2019-12-31

**Authors:** SeungHwan Lee, HanJun Kim, BeomHee Lee

**Affiliations:** 1 Department of Electronic Engineering, Kumoh National Institute of Technology, Gumi, Gyeongbuk 39177, Korea; 2 Automation and Systems Research Institute (ASRI), Department of Electrical and Computer Engineering, Seoul National University, Seoul 08826, Korea; k3k5good@snu.ac.kr (H.K.); bhlee@snu.ac.kr (B.L.)

**Keywords:** SLAM, truncated signed distance, multi-agent SLAM, map merging, rescue system

## Abstract

A novel and an efficient rescue system with a multi-agent simultaneous localization and mapping (SLAM) framework is proposed to reduce the rescue time while rescuing the people trapped inside a burning building. In this study, the truncated signed distance (TSD) based SLAM algorithm is employed to accurately construct a two-dimensional map of the surroundings. For a new and significantly different scenario, information is gathered and the general iterative closest point method (GICP) is directly employed instead of the conventional TSD-SLAM process. Rescuers can utilize a total map created by merging individual maps, allowing them to efficiently search for victims. For online map merging, it is essential to determine the timing of when the individual maps are merged and the extent to which one map reflects the other map, via the weights. In the several experiments conducted, a light-detection and ranging system and an inertial measurement unit were integrated into a smart helmet for rescuers. The results indicated that the map was built more accurately than that obtained using the conventional TSD-SLAM. Additionally, the merged map was built more correctly by determining proper parameters for online map merging. Consequently, the accurate merged map allows rescuers to search for victims efficiently.

## 1. Introduction

It is essential to reduce the rescue time when rescuing the people trapped inside a burning building because the difference between life and death can be a matter of hours. In particular, in disasters such as earthquakes or fires, the goal of search and rescue operations is to rescue the largest number of people in the shortest time, while minimizing risk to rescuers [1]. Location related information and a map of the surroundings are critical for rescuers to navigate the environment safely and to search victims quickly. To provide them with such information, there have been increasing demands for rescue systems or smart helmets in recent years. In particular, the smart helmet with sensor systems or networks [2] can help the rescuers see clearly in the dark during rescue missions. For example, rescuers must keep their hands in contact with the walls or crawl on the ground while carrying the traditional hand-held equipment, which can slow the rescue process. This study proposes a smart helmet as a vital component in the rescue system to search for victims efficiently while building a map of the surrounding environment and localizing the rescuer.

Jeong et al. [3] developed a smart helmet for disaster and safety, which has a novel software framework enabling it to integrate a wide range of devices and services and efficiently manage resources. However, the helmet targets single rescuers instead of a team, with the location of the rescuer being estimated by a global positioning system. Therefore, it is unsuitable for internal environments. The C-Thru smoke diving helmet designed by Haciomeroglu [4] provided a wire frame vision of the interior geometry, helping firefighters identify their surroundings easily and find victims. However, the global localization system is not connected to the helmet. Stefan et al. [5] presented the Hector open source modules for autonomous mapping and navigation with rescue robots. They provided Hector simultaneous localization and mapping (SLAM) modules, which are implemented using an extended Kalman filter and an elevation map, respectively. Their modules were verified in the RoboCup Rescue competition. Nabil et al. [6] conducted rescue missions using two Khepera III robots, called the range-bot and the eye-bot, which were responsible for active SLAM and detecting objects and sending video streams of the objects, respectively. These studies were performed with multi-robot systems, but localization was not considered in detail. Head-SLAM was suggested in [7], which was implemented by GMapping [8,9]. Sensor data were obtained from a two-dimensional (2D) laser scanner and an inertial measurement unit (IMU) on the helmet. The range scan data were projected onto a horizontal plane in the global coordinate system. This approach is similar to the proposed study owing to the application of 2D-SLAM and data projection. However, they did not deal with the multi-agent system for more time-saving strategies. Pascucci et al. [10] suggested an indoor localization framework for hybrid rescue teams using RFID tags. This study focused on a conceptual framework and its performance was verified by the simulation. In [11], wearable technology incorporating novel electrochemical sensors capable of monitoring and detecting the presence of dangerous gases near the firefighter was developed. The authors used a state-of-the-art wireless indoor location tracking system using ultra-wideband (UWB) localization [12] and the hybrid inertial, positional and navigation module. In this study, victims and humans were detected by the UWB and cameras; however, this study focused primarily on online multi-agent SLAM.

Pirkl et al. [13] presented a wearable sensor system that supports construction site workers in work documentation and accesses digital information. This study focused on self-localization using an IMU and light-detection and ranging (LiDAR) in a smart helmet. For a smart helmet, cameras were considered in [14]. The sensors worn by the rescuers were deployed on the helmet, including a GoPro Hero camera and an Xsens AHRS. The helmet highlighted the advantages of a human-robot interaction framework. A team of researchers from Sheffield Robotics invented a helmet fitted with numerous ultrasound sensors used to detect the distances between the helmet and the nearby obstacles [15]. These signals were directly transmitted to vibration pads attached to the inside of the helmet, which were in contact with the wearer′s forehead. Although these systems had their own advantages, they did not consider the SLAM approach; thus, the localization error can significantly increase over time.

Recently, the SLAM approach based on a truncated signed distance function (TSDF) has been proposed for rescue scenarios [16,17,18]. Although the obstacles on the map can be represented accurately using the TSDF, gathering data on a new location can be tedious, e.g., when a door opens or closes abruptly. In addition, it is tedious to apply the TSDF to multi-agent systems directly because all the agents do not share a common coordinate frame and procedure for updating the global map.

To establish a common coordinate frame among the agents, map matching and merging approaches have been investigated [19,20,21,22]. In [19], Carpin used a Hough spectrum to compute the rotation matrix. A translation matrix was computed using the cross-correlation between the *x* and *y* spectra. This approach fitted in indoor environments because lines features were easily extracted in the structured environments. Saeedi et al. [21] improved on the work of Carpin [20] by obtaining more sophisticated translations. Lee et al. [22] proposed a sinogram-based method to simulate offline occupancy grid maps. Sinograms were extracted using Radon transformation which is more accurate but slower than Hough transformation. These studies focused on offline map matching and map merging from the given maps or extended for spaces of higher dimensions [23,24,25].

The present paper proposes a novel and an efficient online rescue scheme that consists of an advanced SLAM framework for a single rescuer and an online Hough spectrum-based map matching and merging method for multiple rescuers. It provides an accurate map to the rescuers using the TSD-based SLAM (TSD-SLAM), in addition to a generalized iterative closest point (GICP) in an abruptly changing environment. Furthermore, the map is a merged map rather than a single map, which helps the rescuers realize which areas have already been covered by others. The proposed framework is connected to a smart helmet equipped with an IMU and a LiDAR sensor through wireless links. In several experiments, rescuers equipped with smart helmets entered test buildings and searched all the rooms using a merged map that was built accurately via the proposed approach.

## 2. Proposed Method for a Single Agent

### 2.1. TSD-SLAM

To cope with dangerous situations, it is important to provide rescuers with accurate maps. A popular map-construction algorithm currently in use is TSD-SLAM, which was developed by Team AutonOHM. It generalizes the Kinect Fusion approach [16]. The TSD-SLAM computes a transformation $T_{k}$ using a model $M_{k}\;=\;\{m_{i}\;|\;i\;=\;1,\ldots ,n_{m}\}$ reconstructed from the TSD grid at the last-known position and a new scene $D_{k}\;=\;\{d_{i}\;|\;i\;=\;1,\ldots ,n_{d}\}$ obtained from the current laser measurements. This is incrementally computed as follows:

(1) $$T_{k}\;=\;T_{k-1}T^{*},\;T^{*}\;=\;\left[\begin{array}{ccc} \cos\alpha & \sin\alpha & t_{x}\\ -\sin\alpha & \cos\alpha & t_{y}\\ 0 & 0 & 1 \end{array}\right],$$

where $T^{*}$ is the transform between $M_{k}$ and $D_{k}$. It consists of a translational vector and a rotation matrix.

For position estimation, the iterative closest point (ICP) algorithm is used together with random sample consensus (RANSAC) based matching [26] for improving the robustness. After the matching process, the truncated signed distance grids $TSDF_{k}\left(x\right)$ at time *k* can be updated as follows:

(2) $$TSDF_{k}\left(x\right)\;=\;\frac{W_{k-1}\left(x\right)\times TSDF_{k-1}\left(x\right)+w_{k}\left(x\right)\times tsdf_{k}\left(x\right)}{W_{k-1}\left(x\right)+w_{k}\left(x\right)},$$

(3) $$W_{k}\left(x\right)\;=\;W_{k-1}\left(x\right)+w_{k}\left(x\right),$$

where $tsdf_{k}\left(x\right)$ represents the truncated signed distance computed at position x in the TSD grid. This update process is similar to the first order low-pass filter. However, because the model was extracted from the TSD grid, the algorithm can deteriorate in situations where a new scene significantly different from the previous one is to be gathered.

### 2.2. Advanced TSD-SLAM

As discussed previously, the TSD-SLAM algorithm is highly useful for constructing complex maps because it generates a model scan from the TSD grid in the matching procedure. However, the SLAM performance with regard to the position and the map accuracy is degraded in reactive situations in which the surrounding environments change significantly. For example, when a rescuer opens a door and enters a room, a significantly different new scene is introduced. In this case, the conventional TSD-SLAM may encounter a matching failure because the existing TSD grid does not reflect the new scene quickly using its weight update process. Subsequently, the TSD grid cannot be updated properly, which may cause the estimated results to diverge. In rescue scenarios, such reactive events occur frequently. To overcome this issue, such events are detected and the GICP method, which is a probabilistic scan-matching method, is used directly [27]. This method is suitable in the case of reactive events, because it only depends on the successive scans and does not require a model extracted from the TSD grid. The event can be detected using Algorithm 1.
**Algorithm 1** Event Detection*DetectionResult* ←*false* Obtain the size $n_{m}$ of model $M_{k}$ $Diff\leftarrow T^{*}{\left[M_{k}\;1_{n_{m}}\right]}^{T}-D_{k,c}{}^{T}$           //Difference between model and corresponding scene
$E_{TF}\leftarrow 0$ **for** all indices *i*
**in**
*Diff*
**do** $E_{TF}\leftarrow \;E_{TF}+sqrt\left(Diff{\left(1,\;i\right)}^{2}+Diff{\left(2,\;i\right)}^{2}\right)$//Accumulated matching error calculation **end for** **If**
$(n_{m}<n_{min}$ | $E_{TF}>E_{TF0})$
**then** *DetectionResult* ←
*true* **end if**

In Algorithm 1, $M_{k}$ and $D_{k,c}$ represent the *k* × 2 matrices. $D_{k,c}$ is obtained from $D_{k}$, which has a set of $n_{m}$ corresponding point pairs from the matching result. Here, $1_{n_{m}}$ represents the $n_{m}$ × 1 matrix filled with 1 s. $n_{min}$ represents the minimum size of the model and $E_{TF0}$ represents the minimum matching error. These values are specified in the point cloud library [28]. If the *DetectionResult* is true, $M_{k}$ is redefined as follows:

(4) $$M_{k}\;=\;D_{k-1\;},$$

where $D_{k-1}$ represents the scene at k−1.

**Figure 1:**
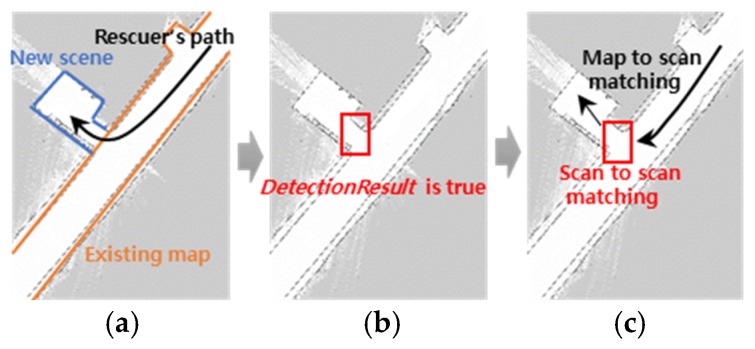
Advanced TSD-SLAM process: (**a**) g*athering* a significantly different new scene; (**b**) d*etecting* the matching failure point; (**c**) scan-to-scan matching is performed instead of map-to-scan matching at the point.

Figure 1 shows the advanced TSD-SLAM process. When a matching failure is detected using the *DetectionResult*, scan-to-scan matching is performed instead of a map-to-scan matching, which is the original TSD process. Scan-to-scan matching describes the GICP. Map-to-scan matching refers to matching between the new scene and the model extracted from the TSD grids.

When $M_{k}$ is aligned with $D_{k}$ using the GICP, the initial coordinates of $M_{k}$ are usually set as a zero vector **0**. However, if the motion of the rescuer changes continuously, the initial coordinates ${\left[x,y,\theta \right]}_{m,0}^{T}$ are represented as follows:

(5) $${\left[\begin{array}{c} x\\ y\\ \theta \end{array}\right]}_{m,0}\;=\;{\left[\begin{array}{c} \Delta t_{k-1,x}\\ \Delta t_{k-1,y}\\ \Delta w_{k,\theta } \end{array}\right]}_{m,0},$$

where $\Delta t_{k-1,x}$ and $\Delta t_{k-1,y}$ represent the *x* and *y* translations, respectively, computed at *k* − 1. $\Delta w_{k,\theta }$ represents the difference between the consecutive yaw angles at *k*. The yaw angles are obtained from the equipped sensors, such as the IMU. Using the initial coordinates, the GICP can converge with high speed and accuracy. Subsequently, the TSD grid can be updated using the TSDF.

## 3. Proposed Method for Multiple Agents

When two or more rescuers enter an unsafe building, they need a common map; that is, their individual maps should be merged. The Hough spectrum-based method [19] as a popular map-matching and map-merging method, is suitable for indoor maps with a large number of lines, such as walls or doors. This is the scenario in most indoor environments. The method can obtain various candidates by finding the maximum cross-correlation in the two spectra and compare them to obtain a more accurate solution. Therefore, the spectrum-based approach was selected for map-matching.

### 3.1. Transform from TSD Grids into Occupancy Grid Map

Map matching algorithms such as the spectrum-based approach used in this study typically depend on the type of the built maps. The type is set as an occupied grid map consisting of free cells, occupied cells, and unknown cells. In the occupancy grid representation, all the cells are populated according to their probability. The TSD grids are transformed into the occupancy grid map as follows:

(6) $$\begin{array}{c} Occ\left(x,y\right)\;=\;\\ \{\begin{array}{c} \mathrm{occupied}\;if\;TSD\left(x\pm 1,y\right)\times TSD\left(x,y\right)<0\;or\;TSD\left(x,y\pm 1\right)\times TSD\left(x,y\right)<0\;\\ \mathrm{free}\;else\;if\;TSD\left(x,y\right)>0\;\\ \mathrm{unknown}\;otherwise\; \end{array} \end{array},$$

where an occupied cell can be identified using the change in the signs of consecutive grids. Free and unknown cells are identified by checking whether TSD(x,y) is positive or negative.

### 3.2. Pre-Processing

**Figure 2:**
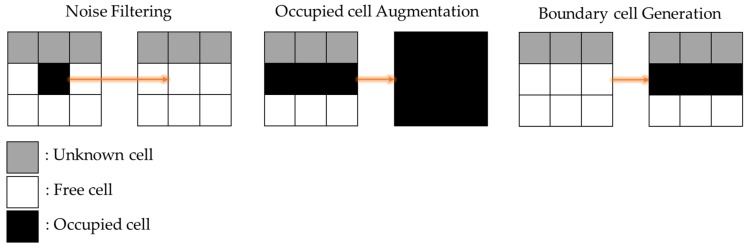
Map pre-processing (including noise filtering, occupied cell augmentation, and boundary cell generation).

To enhance the occupancy map quality, a low-pass filter is applied to the map as shown in Figure 2. Noise can arise owing to the small occupied cells surrounded by the large free cells or vice versa. The cells, which are called the noise, are reclassified via a simple majority vote of the nearest neighbors of each cell. Additionally, to complement the result of the map transform, the occupied cells are augmented and all the boundary cells between the unknown cells and the free cells are assigned to the occupied cells.

### 3.3. Map Merging Algorithm

According to the pre-processing, a high-quality occupancy grid map can be obtained. When two occupancy grids are obtained from two rescuers, the map-matching process can be performed. The occupancy grid maps are input into the Hough spectrum, which is an extension of the Hough transform. A suitable rotation *θ* that produces several solutions can be found between the two occupancy grid maps. After the two maps are aligned using the rotation *θ*, the *x* and *y* spectra are computed to obtain a translation between the two maps. Finally, the map-aligning transform $T_{G}\;=\;{\left[x,y,\theta \right]}^{T}$ is obtained and the occupancy grid maps are merged using the transform as follows:

(7) $$Occ_{M}\left(x,y\right)\;=\;Occ_{1}\left(x,y\right)+T_{G}Occ_{2}\left(x,y\right)\;\mathrm{for}\;\mathrm{all}\;x,y,$$

where $Occ_{M}\left(x,y\right)$ represents the map merged using the two occupancy grid maps. $Occ_{1}\left(x,y\right)$ represents the occupancy grid map of the first rescuer with the reference coordinate system, $Occ_{M}\left(x,y\right)$. $Occ_{2}\left(x,y\right)$ represents the occupancy grid map of the second rescuer, which is transformed using $T_{G}$.

Using the transformation $T_{G}$, the TSD grid of a rescuer can be updated from the other TSD grid. If $TSDF_{k,1}\left(x\right)$ and $TSDF_{k,2}\left(x\right)$ represent the TSD grid of the first and second rescuers, respectively, the two TSD grids can be updated as follows:

(8) $$TSDF_{k,1}\left(x\right)\;=\;w_{T,1}TSDF_{k,1}\left(x\right)+w_{T,2}T_{G}TSDF_{k,2}\left(x\right)\;,\;\;\mathrm{for}\;\mathrm{all}\;x,$$

(9) $$TSDF_{k,2}\left(x\right)\;=\;w_{T,1}TSDF_{k,2}\left(x\right)+w_{T,2}T_{G}^{-1}TSDF_{k,1}\left(x\right),\;\mathrm{for}\;\mathrm{all}\;x,$$

(10) $$w_{T,2}\;=\;1-w_{T,1},$$

where $T_{G}TSDF_{k,2}\left(x\right)$ indicates that all the points in $TSDF_{k,2}\left(x\right)$ are transformed using $T_{G}$. In addition, $T_{G}^{-1}TSDF_{k,1}\left(x\right)$ indicates that all the points in $TSDF_{k,1}\left(x\right)$ are transformed using $T_{G}^{-1}$. As indicated by Equation (8), $TSDF_{k,1}\left(x\right)$ is determined using the sum of $w_{T,1}TSDF_{k,1}\left(x\right)$ and $w_{T,2}T_{G}TSDF_{k,2}\left(x\right).TSDF_{k,2}\left(x\right)$ is also determined by the sum of $w_{T,1}TSDF_{k,2}\left(x\right)$ and $w_{T,2}T_{G}^{-1}TSDF_{k,1}\left(x\right)$. $w_{T,1}$ and $w_{T,2}$ are the weighting factors ranging from 0 to 1.

### 3.4. Timing for Online Map Merging

In the real time approach, the map matching and merging process should be performed at a suitable time. Matching and merging the maps too early may lead to the maps not being merged properly. Additionally, maps may not be merged until the mission is complete. Thus, it is essential to determine when the individual maps are to be merged. The sum of the number of free and occupied cells in an individual occupancy grid map is one of the important criteria for the timing decision. The following inequality should be satisfied by taking the specific threshold β into account:

(11) $$\beta <\frac{\sum_{cell\;=\;free\;or\;occupied}Occ\left(x,y\right)}{gridx\;\times gridy}\times 100\;\left(\%\right),$$

where gridx and gridy denote the *x* and *y* scales of a grid map, respectively. The proper β is empirically determined. If β is too small or large, the maps are not matched completely or not merged until the rescue mission is complete.

If there are several rescuers, Equation (11) should be satisfied for all the occupancy grid maps. Then the map matching process can be performed. However, if the matching quality is low at a certain transform, the transform for map merging cannot be selected. Thus, the overlapped score can be computed as follows:

(12) $$S_{overlapping}\;=\;\frac{agr\left(Occ_{1},\;Occ_{2}\right)}{gridx\;\times gridy},$$

where agr() represents the number of overlapped grids when the two maps are merged via the $T_{G}$. gridx and gridy represent the height and width of the merged map, respectively. $S_{overlapping}$ must to be larger than the empirically defined score $s_{o}$.

$S_{overlapping}$ is the absolute indicator for detecting the matching possibility. In addition, the relative indicator proposed in [20] is employed. The acceptance indicator ai() is defined as:

(13) $$ai\left(Occ_{1},\;Occ_{2}\right)\;=\;\frac{agr\left(Occ_{1},\;Occ_{2}\right)}{agr\left(Occ_{1},\;Occ_{2}\right)+dis\left(Occ_{1},\;Occ_{2}\right)},$$

where dis() represents the number of mismatched grids in the merged map. If $ai\left(Occ_{1},\;Occ_{2}\right)$ is approximately 1.0, there is an overlap between a region of $Occ_{1}$ and a region of $Occ_{2}$; thus, dis() is equal to zero. In [20], it was reported that successful runs had an ai() approximately more than 98% while the ”best” failed attempt had an ai() approximately below 90%. However, in practice, it can be a value below 90%.

### 3.5. Overview of Rescue System

**Figure 3:**
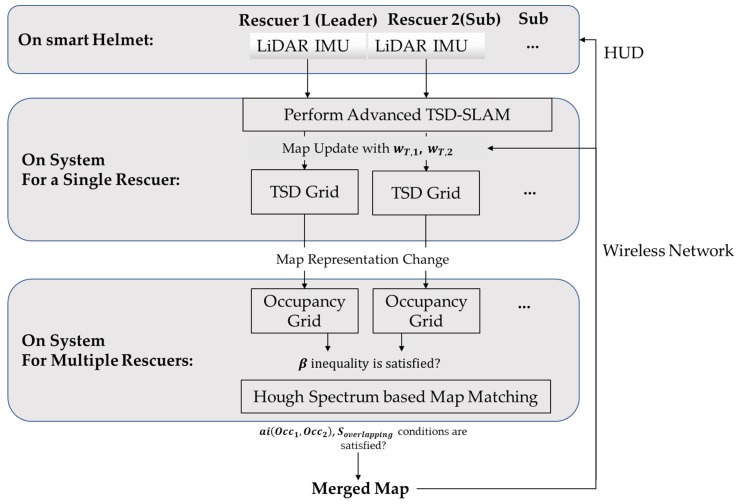
Proposed rescue scheme.

Figure 3 presents the proposed rescue scheme. LiDAR and IMU data for all the rescuers including a lead rescuer are measured using their smart helmets. Using the data, the advanced TSD-SLAM is performed, which estimates the rescuer’s current position and produces a map by updating the TSD grids. At a suitable time to match and merge the maps, the occupancy grid maps are generated using the TSD grid maps. The Hough and *x-y* spectra are generated on the occupancy grid maps and a transformation matrix is produced by determining the local maxima of the spectra cross-correlation. The transformation matrix applies to the individual TSD grid map update, in addition to the occupancy grid map merging process. In the merging process, the map of the leader rescuer is matched and merged with the others one by one. The total map merged from the individual occupancy grid maps is transferred through the wireless network to all the rescuers. Finally, all the rescuers can view the total map in their head-up displays (HUDs). This can help them search for the victims more efficiently.

## 4. Experiments

### 4.1. Smart Helmet

**Figure 4:**
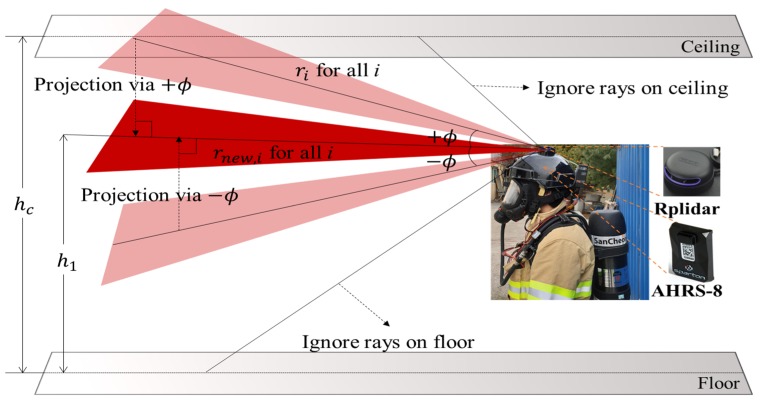
Smart helmet with RPlidar A3 and attitude heading and reference system (AHRS-8), and a projection at the rescuer’s height.

A smart helmet equipped with SLAM Tech’s laser range scanner RPlidar A3 and an attitude heading and reference system (AHRS) was used, as shown in Figure 4. The RPlidar A3 was located at the top of the helmet. The AHRS, which is a three-axis sensor system that provided the real-time three-dimensional (3D) attitude position, was located on the same side. Thus, the 2D scan data ($r_{i},\;\theta_{i})$ in the 3D space were projected onto a plane at the rescuer’s height level $h_{1},$ as follows:

(14) $$r_{new,i}\;=\;r_{i}cos\phi ,\;\mathrm{if}\;\sin^{-1}\left(\frac{h_{1}}{r_{i}}\right)<\phi <\sin^{-1}\left(\frac{h_{c}-h_{1}}{r_{i}}\right)\;\mathrm{for}\mathrm{all}i,$$

where $r_{new,i}$ represents the projected range, $h_{c}$ represents the height of the ceiling from the floor, and ϕ represents the pitch angle. If Equation (14) was not satisfied, $r_{new,i}$ was assigned to an invalid value, indicating that a ray is on either the ceiling or the floor. In the experiments, $h_{1}$ and $h_{c}$ were defined according to the height of the rescuer and the floor-to-ceiling height, respectively. The maximum number of iterations of the GICP and the grid size were set as 100 and 0.05 m, respectively.

### 4.2. Experimental Results

#### 4.2.1. Comparison of Conventional TSD-SLAM and Advanced TSD-SLAM

**Figure 5:**
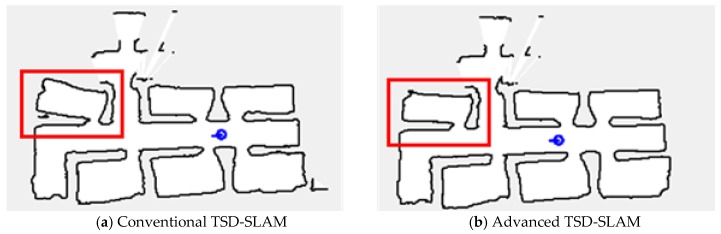
Comparison of the conventional TSD-SLAM and the advanced TSD-SLAM: (**a**) bent map in the red box (matching failure occurs in reactive situations); (**b**) issue resolved.

**Figure 6:**
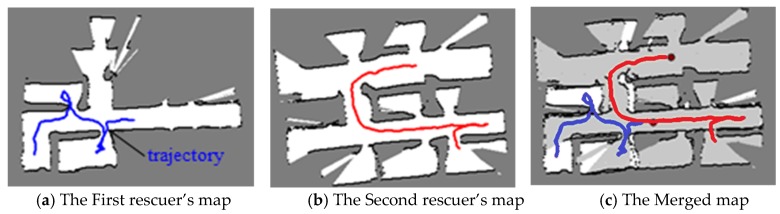
Results for the individual occupancy grid maps and the merged map (the routes of the first and second rescuers are represented by the blue and red lines, respectively).

In the first experiment, the conventional TSD-SLAM and the advanced TSD-SLAM were tested in test bed environments (approximately 14 m × 14 m in size). The performance of the advanced TSD-SLAM is shown in Figure 5. The rooms in the red boxes in the map of Figure 5a,b were built differently. However, they corresponded to the same room. In the conventional TSD-SLAM, there were orientation errors as the door was opened. However, the orientation was estimated correctly via the advanced TSD-SLAM, as shown in Figure 5b. Finally, the merged map was obtained via the proposed rescue scheme, which enabled the rescuers to choose non-overlapped paths, as shown in Figure 6. The TSD grid update was not considered after the map matching process. Thus, multiple overlapping lines can represent one wall. The overlapped lines can end up with distorting results such as mapping failures.

#### 4.2.2. Consideration of TSD Grid Update

**Figure 7:**
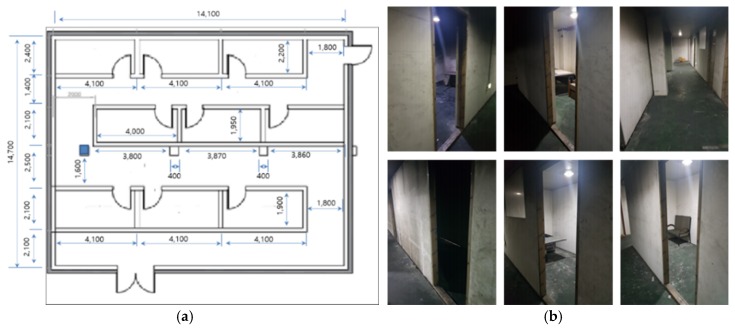
Test indoor environment: (**a**) top view of the environment; (**b**) all the rooms and corridors were basic in design).

**Figure 8:**
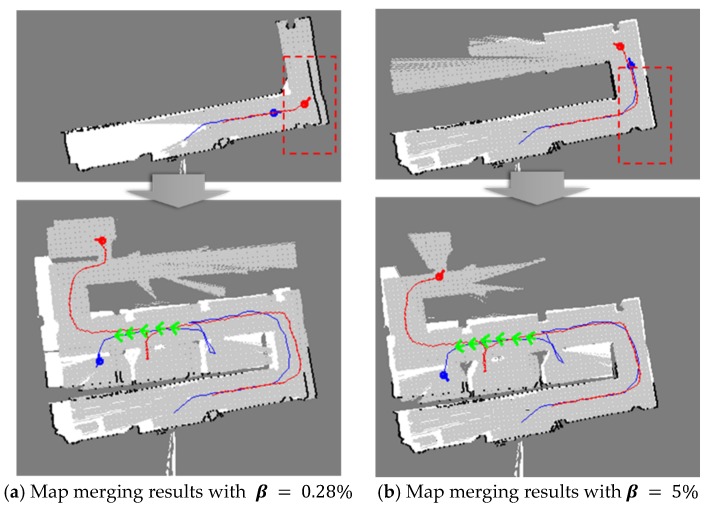
Qualitative comparison of the merged maps according to β.

In the second experiment, the artificial walls were moved and all the rooms in the building were rearranged as shown in Figure 7. The size of the test indoor environment was approximately 14 m × 14 m. There were several rooms, corridors, and walls. The people who played the role of victims stayed in certain rooms. First, β was changed in the tests in order to determine the proper β. Figure 8 shows the test results. When β was very small, i.e., β = 0.28%, the maps were not aligned well because there were many candidates for merging. However, when β was set as 5%, the maps were merged more accurately. In several experiments, β was determined, ranging from 2% to 5% because typical buildings have only one entrance. If there are several entrances, β should be large enough to search the same structure in the maps. Two rescuers using three different methods built the whole map. The map merging process was included in the first method without any update procedures. In the second and the third methods, the TSD grids were updated after the map merging, however, the weight, $w_{T,1}$ was different. In the tests, $w_{T,1}$ was set as 0.95 in the second method and 0.5 in the third method, respectively.

**Figure 9:**
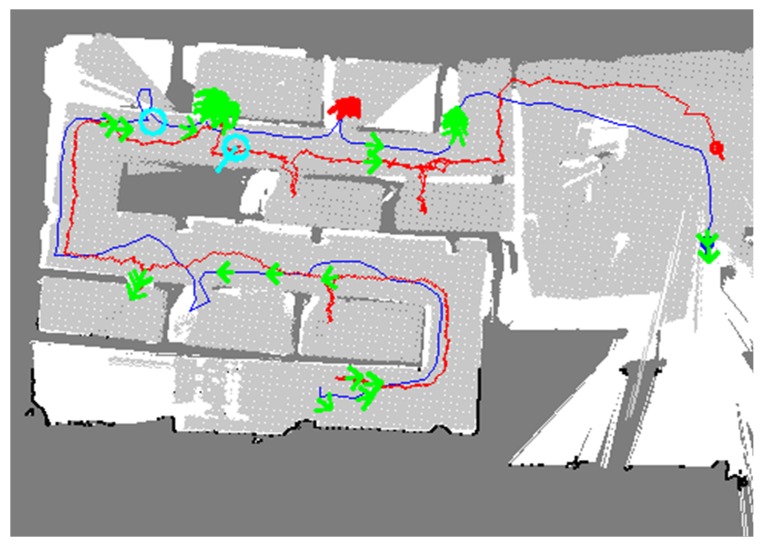
Map merging result without the TSD grid update; the two maps were imperfectly merged.

Figure 9 shows the result of the first method. The blue and red lines represent the trajectories of rescuers 1 and 2, respectively. The green arrows represent the UWB results, and the cyan arrows represent the results for the detection of humans and victims in the room. The first method, however, did not update the individual TSD grids even after the occupancy grid maps were merged. In the merged map, it was evident that the two occupancy grid maps are different in terms of the free cells represented by the white and light grey colors and it was difficult to understand the whole map correctly.

**Figure 10:**
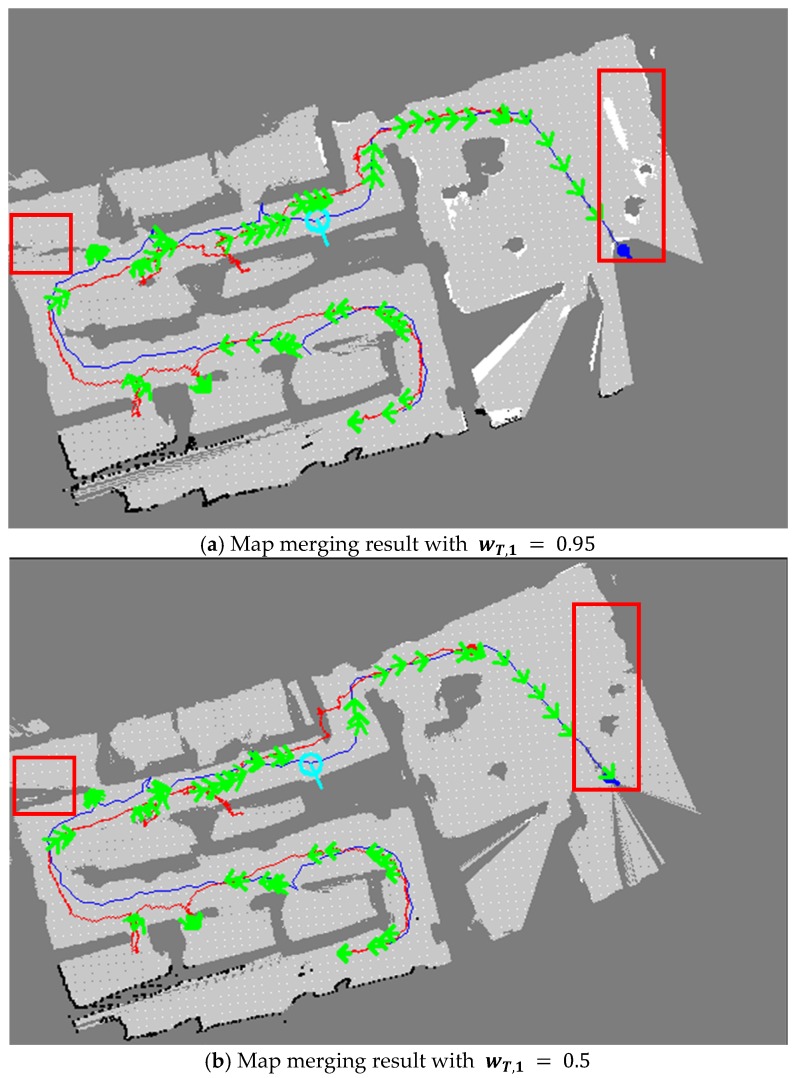
Map merging results with the TSD grid update. By comparing the two maps, a more correct map was built as shown in (**b**).

With regard to the TSD grid update, the second and third methods performed better than the first method. Figure 10 shows the map merging results of the second and third methods. As the second method had a low ratio of one TSD grid being reflected in the other TSD grid update, several parts did not overlap perfectly, as shown in Figure 10a. However, the merged map in the third method is apparently represented as shown in Figure 10b. Thus, in the third approach, the merged map was more accurately built by comparing several parts with other methods.

**Figure 11:**
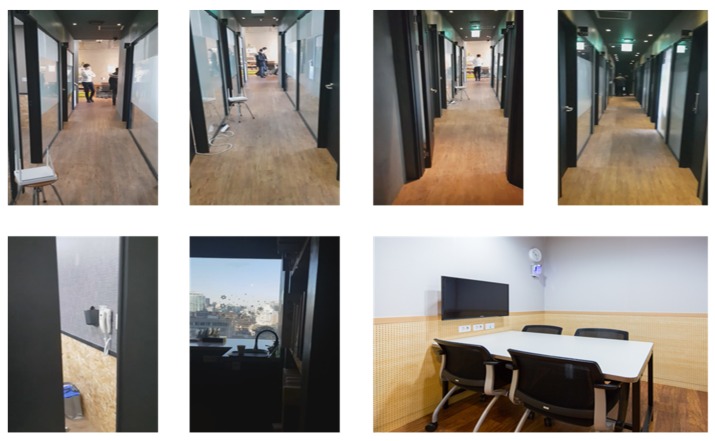
Indoor environment for the experiments (meeting room).

**Figure 12:**
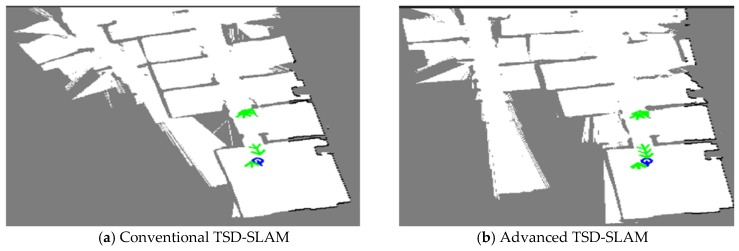
Comparison of the conventional TSD-SLAM and the advanced TSD-SLAM. The left part of the map was not built accurately in (**a**), but the matching errors were reduced in (**b**).

The third experiment was conducted in a public indoor environment with many meeting rooms, as shown in Figure 11. In contrast to the second environment, it consisted of glass doors and windows, which may cause errors in the SLAM. First, the performance of the advanced TSD-SLAM was compared with that of the conventional one, as shown in Figure 12. In particular, the left part of the map was built accurately as shown in Figure 12b, whereas the conventional TSD-SLAM accumulated orientation errors over time. The difference between results of the conventional and advanced TSD-SLAM methods was clearly observed in the environment with many rooms.

**Figure 13:**
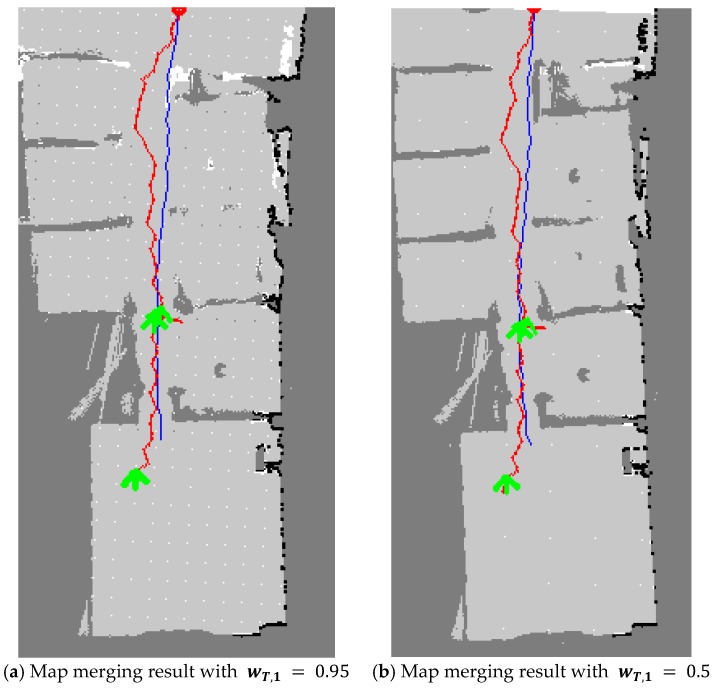
Map merging results with the TSD grid update. By comparing the two maps, a more accurate map was built, as shown in (**b**).

Figure 13 shows the comparison results. The quality of the merged map with the TSD grid update using a large weight $w_{T,1}$, was low, indicating that it is tedious to reduce the errors that occur in the SLAM. This is because one rescuer’s TSD grid map rarely affects the other rescuer’s TSD grid map, as shown in Figure 13a. In contrast, if the maps are merged using the TSD grid update process with a weight $w_{T,1}$ equal to the other weight $w_{T,2}$, a more accurate map can be obtained, as shown in Figure 13b. As indicated by the figures, the errors that occurred in the SLAM were significantly reduced. Thus, an individual SLAM can be enhanced using the results of accurate map merging.

**Figure 14:**
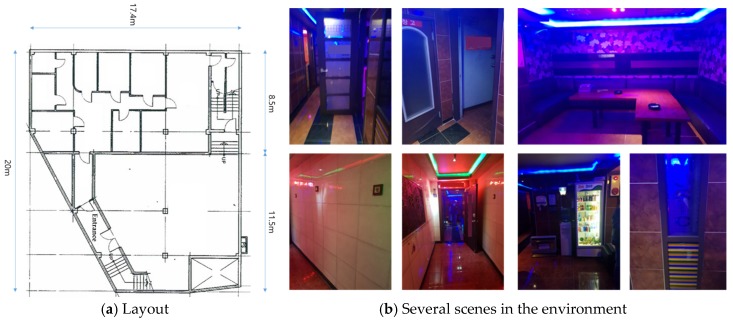
Indoor environment for the experiments.

**Figure 15:**
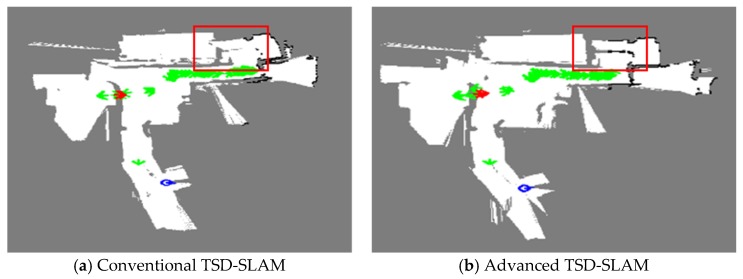
Comparison of the conventional TSD-SLAM and the advanced TSD-SLAM: (**a**) bent map in the red box (matching failure occurs in reactive situations); (**b**) issue resolved.

In the fourth experiment, a karaoke venue called Noraebang in Korea was tested, as shown in Figure 14. It consisted of several rooms with glass doors and different floor-to-ceiling heights, which could induce errors in the SLAM. Figure 14a shows the blueprint of the experimental environment; it was approximately 18 m × 18 m in size. The performance of the advanced TSD-SLAM is shown in Figure 15. In the conventional TSD-SLAM, (Figure 15a) there were orientation errors, particularly, in the area marked by the red box. However, the orientation was estimated correctly in Figure 15b even though the rescuers frequently opened and closed doors to search for victims.

**Figure 16:**
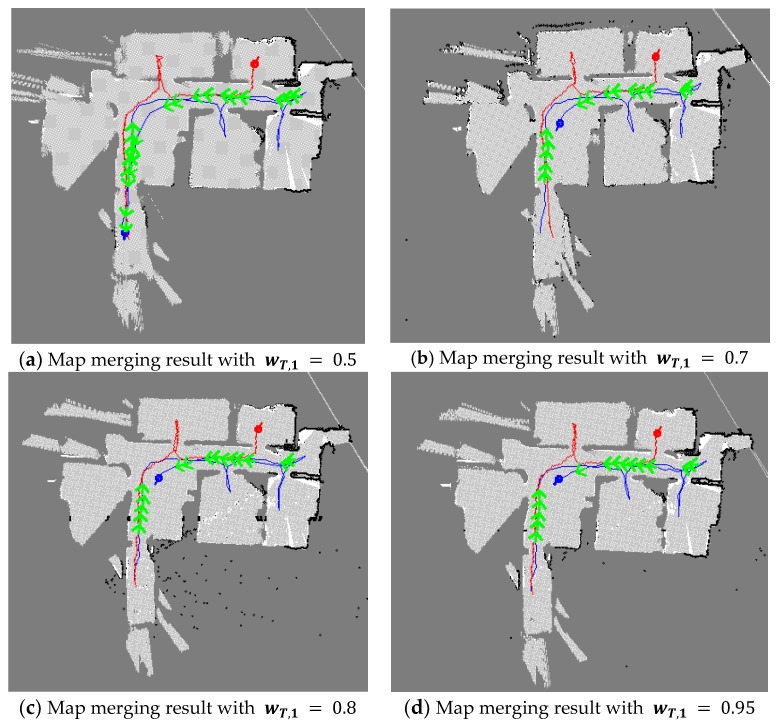
Map merging result with the TSD grid update (when there are errors in map-matching, it is beneficial to update the merged map with a larger weight $w_{T,1}$).

Figure 16 shows the comparative results where the shape of the rooms can be recognized in the map merging result. However, in some areas, the rooms and the corridors were not separated. Additionally, errors due to the matching or the update were observed. As shown in Figure 16b,c, there were matching errors, which were not removed until the end of the experiment. However, in Figure 16d, all the rooms can be easily recognized and clearly separated. This indicates that if there are errors in map matching, it is more beneficial to update the merged map with a larger weight $w_{T,1}$ than to use a weight $w_{T,1}$ equal to the other weight $w_{T,2}$.

### 4.3. Discussion: Analysis of Results

In the several indoor environments, the proposed approach was verified by comparing it with the conventional TSD SLAM and by changing parameters such as, $w_{T,1}\;\mathrm{and}\;w_{T,2}$. With regard to the SLAM result, a more accurate map was achieved when a rescuer abruptly opened or closed the doors, thereby reducing the orientation error. In the map merging, it was beneficial to update the TSD grid with $w_{T,1}\;=\;0.5$, indicating that the other map information was highly reflected in the map update. However, if errors occur in a complex environment before and after the map matching, it is advisable to assign $w_{T,1}$ with a larger weight. In the map comparison, it was complex to show the quantity evaluation because a global reference system could not be applied. Nevertheless, the methods were compared graphically and analyzed. To obtain an accurate map, the timing of the online map merging was critical. Table 1 presents the parameters used in all the experiments. β was the criterion for the map merging. As mentioned previously, it ranged from 2% to 5%. In experiments 1 and 2, β was relatively large because it was beneficial to take more time for accurate map merging rather than to increase the individual SLAM accuracy resulting from the map-merging. However, in experiments 3 and 4, β decreased because the environments were complex, and the SLAM could fail. Once the criterion β was satisfied, the two values for ai() and $S_{overlapping}$ were checked, which were closely related to the map merging quality. ai() was set as 0.95, in accordance with [20]. A larger value can ensure the map merging quality. $S_{overlapping}$ was set as 0.03, which was used for the minimum criterion. When the grid number of the merged map was 360,000 (600 × 600), more than 11,000 matched grids satisfied this criterion. In the complex environments, i.e., experiments 3 and 4, since the SLAM could fail, ai() and $S_{overlapping}$ were reduced to 0.85 and 0.02, respectively. If we reduced these values significantly, the quality of map merging could not be ensured. All the experimental results indicated that the maps were merged properly in real-time.

## 5. Conclusions

We propose a novel and an efficient rescue scheme using the online multi-agent SLAM framework. In a rescue scenario, the proposed framework provides the rescuers with accurate maps. Technically, it produces a merged map to avoid the overlapping paths of individual maps. This reduces time and saves lives. Several experiments were performed using smart helmets equipped with IMU and LiDAR sensors. The sensors were connected to the proposed framework via a wireless network. The results indicated that the performance of the proposed method was superior to that of the conventional TSD-SLAM, with regard to the map-construction accuracy. The map was constructed accurately even when a significantly different new scene was to be gathered. The merged map was obtained via a Hough-spectrum based method by transforming the TSD grids into an occupancy grid map. For online map merging, the time when the individual maps were merged, and the amount of one map reflected in the other map via the weights, were determined. In several experiments, the proposed approach exhibited significantly higher performance than others, and suitable parameters for obtaining a more accurate map were discovered.

## Figures and Tables

**Table 1:** Parameters for online map merging.

Parameters	Experiment 1	Experiment 2	Experiment 3	Experiment 4
β	5%	5%	4%	2.5%
ai()	0.95	0.95	0.85	0.85
$S_{overlapping}$	0.03	0.03	0.02	0.02
